# Association of Oxidative Stress With Biochemical Markers in Diabetic and Non-diabetic Chronic Kidney Disease Patients: A Cross-Sectional Study

**DOI:** 10.7759/cureus.102974

**Published:** 2026-02-04

**Authors:** Manpreet Saini, Vijay Kumar, Jaun Z Rizvi

**Affiliations:** 1 Biochemistry, Laxmi Chandravansi Medical College and Hospital, Palamu, IND; 2 Community Medicine, Laxmi Chandravansi Medical College and Hospital, Palamu, IND; 3 Community Medicine, Autonomous State Medical College, Lakhimpur Kheri, IND

**Keywords:** chronic kidney disease, diabetes, diabetic kidney disease, mda, oxidative stress

## Abstract

Background

Chronic kidney disease (CKD) is exacerbated by oxidative stress (OS), which accelerates renal injury and cardiovascular complications. Diabetes intensifies OS via hyperglycemia and mitochondrial dysfunction, promoting faster decline in diabetic kidney disease (DKD). This study compares OS markers with standard biochemical parameters in diabetic versus non-diabetic CKD to inform risk stratification and management.

Methods

This hospital-based, cross-sectional study enrolled 240 CKD patients (108 diabetic, 132 non-diabetic) over one year. Demographics, clinical data, and biochemical parameters-including fasting/postprandial blood sugar (FBS/PPBS), HbA1c, creatinine, urea, uric acid, estimated glomerular filtration rate (eGFR), and malondialdehyde (MDA)-were collected. Group comparisons, ANOVA, post-hoc Tukey, and Pearson's correlation assessed associations, with p<0.05 considered significant.

Results

A total of 240 patients with chronic kidney disease were included in the study, of whom 108 (45.0%) had DKD and 132 (55.0%) had non-DKD. Most patients, 135 (56.2%), were aged 40-59 years. Regarding sex distribution, 163 (67.9%) were male, and 77 (32.1%) were female. Stage 5 CKD was the most prevalent, observed in 142 (59.2%) patients. Patients with DKD demonstrated significantly higher FBS, PPBS, HbA1c, uric acid, and MDA, with lower eGFR compared to non-DKD (p<0.05). MDA levels showed a positive correlation with blood urea, serum creatinine, uric acid, and CKD stage, and a negative correlation with eGFR (p<0.05).

Conclusion

Diabetic CKD patients exhibit a higher oxidative burden than non-diabetic CKD patients, and MDA levels rise progressively with worsening CKD stage. These results emphasize the clinical relevance of oxidative stress in renal dysfunction and suggest its potential role as a prognostic biomarker and therapeutic target in CKD.

## Introduction

Chronic kidney disease (CKD) is a progressive disorder in which oxidative stress (OS) is considered to play an important role. OS results from an imbalance between reactive oxygen species and antioxidant defenses, leading to tubular damage, glomerulosclerosis, and cardiovascular complications [1, 2]. In diabetes, persistent hyperglycemia, advanced glycation end products, and mitochondrial dysfunction further intensify OS, which helps explain the faster progression of diabetic kidney disease (DKD) compared with non-diabetic CKD [3]. Several reviews and cohort studies have shown that OS is not only a consequence of renal impairment but also an active driver of disease progression and extra-renal complications [1, 4].

Various biomarkers have been used to assess oxidative stress in both CKD and diabetes. These include lipid peroxidation products such as malondialdehyde (MDA) and F2-isoprostanes, DNA damage markers such as 8-hydroxyguanosine (8-OHG), 8-hydroxy-2′-deoxyguanosine (8-OHdG), protein oxidation products, and antioxidant enzymes like superoxide dismutase, catalase, and glutathione peroxidase [2, 5]. At the same time, standard biochemical markers - serum creatinine, blood urea, uric acid, estimated glomerular filtration rate (eGFR), and albuminuria - remain the cornerstone for CKD staging and monitoring [6]. However, the direct relationship between oxidative stress and these biochemical parameters in diabetic and non-diabetic CKD has not been fully established. Recent cross-sectional and population-based studies have also shown that indices such as the oxidative balance score (OBS) are associated with CKD severity, emphasizing the importance of systemic redox status [7].

Other factors unique to CKD further amplify oxidative stress. Protein-bound uremic toxins such as indoxyl sulfate and p-cresyl sulfate, derived from gut microbial metabolism, are inversely correlated with eGFR and promote inflammation, endothelial dysfunction, and oxidative imbalance [8, 9]. Modern imaging techniques now allow in vivo visualization of renal oxidative stress, providing stronger evidence of its role in disease progression [10].

Among the available biomarkers, MDA is widely used as a commonly accepted measure of lipid peroxidation. It is simple and cost-effective to measure using the thiobarbituric acid reactive substances (TBARS) method and has been consistently linked with CKD progression and cardiovascular complications. Its practicality and reproducibility make it suitable for large hospital-based studies.

Despite increasing evidence, few studies have compared oxidative stress between diabetic and non-diabetic CKD patients in relation to routine biochemical markers. Therefore, the objective of this study was to compare oxidative stress levels, measured by serum MDA, between diabetic and non-diabetic patients with chronic kidney disease. A secondary objective was to evaluate the association between MDA levels and routine biochemical parameters, including renal function indices, across different stages of CKD.

## Materials and methods

This hospital-based, cross-sectional, comparative study was carried out over a period of one year, from June 2020 to June 2021, on patients diagnosed with chronic kidney disease (CKD). The study population comprised CKD patients admitted to or attending the outpatient departments of various tertiary care hospitals in Lucknow, India. Participants were recruited using a consecutive sampling technique during the study period without restrictions on age or gender. Prior approval was obtained from the Institutional Ethics Committee of Malwanchal University, Indore, Madhya Pradesh, India (MU/Research/EC/Ph.D/2020/04). Written informed consent was obtained from all patients after explaining the study objectives, the voluntary nature of participation, and their right to withdraw at any time without affecting their medical care. No financial incentives were provided, and routine clinical management continued regardless of participation. A total of 240 CKD patients were enrolled after consent. Among them, 108 were classified as diabetic CKD (DKD) patients, defined by the presence of chronic kidney disease along with diabetes mellitus, diagnosed according to American Diabetes Association (ADA) criteria based on elevated blood glucose and HbA1c levels. The remaining 132 were non-diabetic CKD (non-DKD) patients, confirmed by abnormal kidney function tests with normal glucose and HbA1c levels. Inclusion criteria comprised patients of either sex, aged 18 years or above, with confirmed CKD with or without diabetes. Exclusion criteria included pregnancy, sepsis, acute febrile illness, critical illness, or CKD patients receiving glucocorticoids, β-blockers, or oral contraceptives. Patients with a past history of diabetes but with normal glycemic and HbA1c values at the time of enrollment were also excluded.

Data were collected using a pre-structured proforma that recorded demographic information (age, sex), clinical details, and laboratory results. Fasting venous blood samples were collected under aseptic conditions and analyzed for fasting blood glucose (FBG), serum creatinine, blood urea, serum uric acid, estimated glomerular filtration rate (eGFR), and serum malondialdehyde (MDA). Serum samples for malondialdehyde estimation were separated immediately after centrifugation and analyzed on the same day to minimize oxidative degradation. Where short delays occurred, samples were stored at 2-8°C and analyzed within 24 hours. Two hours after a standard meal, postprandial blood samples were obtained from the same participants for postprandial blood sugar (PPBS) measurement. Biochemical analyses were performed using standard methods. FBG and PPBS were measured using the glucose oxidase-peroxidase (GOD-POD) method. HbA1c was estimated by ion-exchange high-performance liquid chromatography (HPLC). Serum creatinine was determined by Jaffe's method, blood urea by the modified Berthelot method, and serum uric acid by the uricase method. eGFR was calculated using the CKD-EPI equation. Lipid peroxidation marker MDA was assessed by the thiobarbituric acid reactive substances (TBARS) method. All samples were analyzed in duplicate using standard internal quality control procedures. Automated analyzers were used for all assays: an ELICO UV-visible spectrophotometer (ELICO Ltd., Hyderabad, India) for malondialdehyde estimation, a Bio-Rad D-10 analyzer (Bio-Rad Laboratories, Hercules, CA, USA) for HbA1c measurement, and a Roche Cobas 6000 c501 analyzer (Roche Diagnostics, Mannheim, Germany) for other biochemical parameters. Data were entered into Microsoft Excel (Microsoft, Redmond, Washington) using a predesigned template and subsequently analyzed with SPSS version 15.0 (IBM Inc., Armonk, New York). Categorical variables such as age distribution, gender distribution, and CKD stage were expressed as frequencies and percentages. Continuous variables were presented as mean ± standard deviation and range. Intergroup comparisons were performed using the Chi-squared test for categorical variables and Student's t-test for continuous variables. ANOVA was used to analyze differences in mean MDA levels among different CKD stages, and significant values were followed by a post-hoc Tukey test. A p-value of <0.05 was considered statistically significant. Correlation between MDA and biochemical parameters was analyzed using Pearson's correlation coefficient, with statistical significance set at p<0.01. Normality of continuous variables was assessed prior to applying parametric tests. Given the exploratory and cross-sectional nature of the study, potential confounding factors were addressed through stratified group comparisons.

## Results

A total of 240 patients with CKD were included in the study. Table 1 summarizes the demographic and clinical characteristics of the study population, comparing diabetic CKD (DKD) and non-diabetic CKD (non-DKD) patients. Of the total 240 CKD patients included, 108 (45.0%) had DKD and 132 (55.0%) had non-DKD. Most patients were aged 40-59 years (135, 56.2%), followed by those aged ≥60 years (69, 28.8%) and 20-39 years (36, 15.0%). A statistically significant difference in age distribution was observed between DKD and non-DKD patients, with DKD patients being older overall (p<0.001). Male patients predominated in the study population, accounting for 163 (67.9%) cases, with a similar male predominance observed in both DKD and non-DKD groups. The difference in sex distribution between the two groups was not statistically significant (p=0.922). With respect to CKD staging, most patients were in advanced stages of the disease; 142 (59.2%) were in stage 5, followed by 75 (31.2%) and 23 (9.6%) in stage 4 and 3, respectively. Stage 5 CKD was more frequent among DKD patients (69, 63.9%) than among non-DKD patients (73, 55.3%); however, the overall distribution of CKD stages between the two groups was not statistically significant (p=0.129).

**Table 1:** Demographic distribution of diabetic and non-diabetic CKD patients CKD - chronic kidney disease

Variable	Category	Diabetic CKD, n (%)	Non-diabetic CKD, n (%)	Total	χ2	p-value
Age (years)	20-39	4 (3.7)	32 (24.2)	36 (15.0)	19.65	<0.001
	40-59	69 (63.9)	66 (50.0)	135 (56.2)		
	≥60	35 (32.4)	34 (25.8)	69 (28.8)		
Sex	Male	73 (67.6)	90 (68.2)	163 (67.9)	0.009	0.922
	Female	35 (32.4)	42 (31.8)	77 (32.1)		
CKD stage	Stage 3	6 (5.6)	17 (12.9)	23 (9.6)	4.094	0.129
	Stage 4	33 (30.6)	42 (31.8)	75 (31.2)		
	Stage 5	69 (63.9)	73 (55.3)	142 (59.2)		

Table 2 compares oxidative stress and biochemical parameters among DKD and non-DKD patients. As expected, glycemic indices were markedly elevated in the diabetic group, with significantly higher mean FBS (216.05 ± 60.08 vs. 91.61 ± 10.84 mg/dl, p<0.001), PPBS (307.48 ± 81.30 vs. 102.92 ± 14.54 mg/dl, p<0.001), and HbA1c (9.32 ± 2.10 vs. 4.80 ± 0.39%, p<0.001). Uric acid was also significantly higher in DKD patients (11.19 ± 3.62 vs. 7.51 ± 2.22 mg/dl, p<0.001). Conversely, mean eGFR was significantly lower in diabetics (13.06 ± 8.10 vs. 16.42 ± 11.86 ml/min/1.73m², p=0.013). No significant differences were observed in blood urea (p=0.825) and serum creatinine (p=0.294). Importantly, oxidative stress marker MDA was substantially elevated in diabetics (9.72 ± 2.78 vs. 5.20 ± 1.51 nmol/ml, p<0.001), highlighting a strong association between diabetes, CKD progression, and oxidative imbalance.

**Table 2:** Comparison of oxidative stress and other biochemical parameters among DKD and non-DKD patients DKD - diabetic chronic kidney disease; FBS - fasting blood sugar; PPBS - postprandial blood sugar; eGFR - estimated glomerular filtration rate; MDA - malondialdehyde

Variable (mg/dl)	DKD (n=108)		Non-DKD (n=132)		t-value	p-value
	Range	Mean±SD	Range	Mean±SD		
FBS	121-373	216.05±60.08	72-118	91.61±10.84	23.345	<0.001
PPBS	189-548	307.48±81.30	72-138	102.92±14.54	28.371	<0.001
HbA1c (%)	4.5-14.1	9.32±2.10	4.0-5.7	4.80±0.39	24.186	<0.001
Blood urea	47-304	112.72±58.03	21-247	114.37±56.88	-0.221	0.825
Serum creatinine	2-13.8	5.79±2.91	1.1-15.2	5.38±3.16	1.051	0.294
Uric acid	3.7-17.3	11.19±3.62	3.2-13.2	7.51±2.22	9.650	<0.001
eGFR (ml/min/1.73m^2^)	3-35	13.06±8.10	3-58	16.42±11.86	-2.504	0.013
MDA (nmol/ml)	3.7-15.9	9.72±2.78	1.5-8.0	5.20±1.51	15.999	<0.001

Table 3 presents the Pearson's correlation of oxidative stress marker MDA with biochemical parameters in diabetic and non-DKD patients. In DKD patients, MDA showed strong positive correlations with blood urea (r=0.680, p<0.001), serum creatinine (r=0.806, p<0.001), uric acid (r=0.728, p<0.001), and CKD stage (r=0.705, p<0.001). A strong inverse correlation was noted with eGFR (r=−0.810, p=0.000). Similar but slightly weaker correlations were observed in non-DKD patients, where MDA correlated positively with blood urea (r=0.572, p<0.001), serum creatinine (r=0.661, p<0.001), uric acid (r=0.567, p<0.001), and CKD stage (r=0.702, p<0.001), and inversely with eGFR (r=−0.701, p<0.001). No significant correlations were found with age or glycemic indices (FBS, PPBS, HbA1c) in either group, except a weak negative correlation of MDA with PPBS in diabetics (r=−0.235, p=0.014).

**Table 3:** Correlation between MDA and other parameters in DKD and non-DKD patients DKD - diabetic chronic kidney disease; CKD - chronic kidney disease; FBS - fasting blood sugar; PPBS - postprandial blood sugar; eGFR - estimated glomerular filtration rate; MDA - malondialdehyde

Parameter	MDA in Diabetic CKD		MDA in non-diabetic CKD	
	Pearson correlation	p-value	Pearson correlation	p-value
Age	-0.079	0.415	0.045	0.606
FBS	-0.166	0.086	-0.116	0.185
PPBS	-0.235	0.014	-0.068	0.437
HbA1c	-0.121	0.213	0.169	0.052
Blood urea	0.680	<0.001	0.572	<0.001
Serum Creatinine	0.806	<0.001	0.661	<0.001
eGFR	-0.810	<0.001	-0.701	<0.001
Uric acid	0.728	<0.001	0.567	<0.001
CKD stage	0.705	<0.001	0.702	<0.001

Table 4 shows the mean MDA levels across different CKD stages. A clear, progressive increase in oxidative stress was observed with advancing CKD stage. Mean MDA was lowest in stage 3 (4.06 ± 1.28 nmol/ml), higher in stage 4 (5.60 ± 2.17 nmol/ml), and highest in Stage 5 (8.61 ± 2.98 nmol/ml). One-way ANOVA demonstrated a highly significant overall difference in MDA levels across the different stages of CKD (f=9.89, p<0.001). Post-hoc Tukey analysis revealed that MDA levels differed significantly between all stage pairs: stage 3 versus stage 4 (p=0.016), stage 4 versus stage 5 (p<0.001), and stage 3 versus stage 5 (p<0.001). These results indicate a stepwise and progressive increase in oxidative stress with worsening CKD severity.

**Table 4:** MDA levels across CKD stages with post-hoc comparisons CKD - chronic kidney disease; MDA - malondialdehyde

CKD stage	MDA (nmol/ml) range	Mean ± SD	Stage comparisons	p-value
Stage 3	1.9-8.8	4.06 ± 1.28	Stage 3 vs stage 4	0.016
			Stage 3 vs stage 5	<0.001
Stage 4	1.5-10.1	5.60 ± 2.17	Stage 4 vs stage 5	<0.001
Stage 5	3.8-15.9	8.61 ± 2.98	-	-

## Discussion

In this cross-sectional study, we evaluated oxidative stress and its association with biochemical markers in 240 patients with chronic kidney disease (CKD), comparing diabetic (n=108) and non-diabetic (n=132) cohorts. The majority of participants were male and belonged to the 40-59-year age group, consistent with an earlier report by Bikbov et al. describing male predominance and a higher burden of CKD in middle-aged adults [11]. Advanced CKD (stage 5) was the most frequently observed stage in both groups, reflecting delayed presentation commonly reported in resource-limited healthcare settings as described by Levey et al. and Afkarian et al. [6, 12]. Patients with diabetic kidney disease (DKD) were older than their non-diabetic counterparts, supporting previous observations by Zhao et al. that diabetic nephropathy typically presents later but progresses more rapidly [13]. As expected, glycemic indices were significantly higher in the DKD group, confirming the metabolic dysregulation inherent to diabetes. These findings align with observations by Duni et al., who emphasized hyperglycemia as a key driver of oxidative stress and renal injury through mechanisms such as advanced glycation end-product formation and mitochondrial overproduction of reactive oxygen species (ROS) [1]. Although blood urea and serum creatinine levels did not differ significantly between groups, serum uric acid levels were markedly higher in DKD patients. This supports growing evidence, as reported by Wu et al. and Su et al., that uric acid functions not only as a biomarker but also as a mediator of oxidative stress and renal damage in CKD [14, 15].

Malondialdehyde (MDA), a well-established marker of lipid peroxidation, was significantly elevated in DKD patients compared to non-DKD patients. This finding corroborates previous studies by Ling et al. [16] and Adeshara et al. [17], demonstrating enhanced oxidative stress in diabetic nephropathy due to the cumulative effects of hyperglycemia, chronic inflammation, and impaired antioxidant defenses. The strong positive correlations observed between MDA and blood urea, serum creatinine, uric acid, and CKD stage, along with its inverse relationship with estimated glomerular filtration rate (eGFR), highlight the close association between oxidative imbalance and declining renal function. Similar correlation patterns have been reported previously; however, the stronger associations observed in diabetic patients in our study suggest that diabetes may amplify oxidative renal injury as found by Zhao et al. and Wang et al. [13, 18].

Stage-wise analysis revealed a progressive increase in MDA levels from CKD stage 3 to stage 5, with post-hoc comparisons confirming statistically significant differences between all stages. These findings are consistent with longitudinal studies reported by Daenen et al., reporting stepwise increases in oxidative stress as CKD advances [5]. Notably, diabetic patients demonstrated higher oxidative stress at each CKD stage, supporting the concept that hyperglycemia-induced metabolic disturbances accelerate ROS generation and compromise renal antioxidant capacity, as highlighted by Zhao et al. [13]. The absence of a strong correlation between MDA and glycemic indices, except for a weak negative association with postprandial blood sugar in diabetic patients, suggests that chronic oxidative stress in CKD may be driven more by renal dysfunction and accumulation of uremic toxins than by short-term glycemic variability, as suggested by a recent study [17]. Collectively, these findings highlight the complex interplay between metabolic abnormalities, renal impairment, and oxidative stress, emphasizing the importance of an integrated biochemical assessment in patients with CKD.

This study's strengths include its clinically relevant focus on oxidative stress in diabetic and non-diabetic CKD, use of standardized biochemical methods and CKD staging, a relatively large sample size, and stage-wise analysis. Automated assays and routine hospital care helped minimize variability and potential confounders. Limitations include the predominance of advanced CKD and the lack of multivariable analysis. Age differences reflect the later onset of diabetic nephropathy. Oxidative stress was assessed solely via MDA using the TBARS assay at a single time point, which does not capture the full or longitudinal oxidative profile. Despite these limitations, the study provides meaningful comparative insights and a basis for future research.

## Conclusions

Oxidative stress, reflected by elevated malondialdehyde (MDA) levels, was significantly higher in patients with diabetic kidney disease compared to those with non-diabetic CKD and increased progressively with advancing stages of chronic kidney disease. The observed correlations between MDA and renal function parameters demonstrate an association between oxidative imbalance and worsening renal dysfunction, particularly in the diabetic setting. These findings indicate that oxidative stress is closely linked with CKD severity. Monitoring oxidative stress markers alongside routine biochemical parameters may help in assessing disease burden and guiding individualized management. While interventions targeting oxidative stress - such as optimized glycemic control, lifestyle modification, or antioxidant-based therapies - may be considered, their effects on disease progression and cardiovascular risk remain to be confirmed. Further prospective studies are needed to clarify the role of targeted strategies in improving outcomes among patients with diabetic CKD.
